# A methodological investigation of healthy tissue, hepatocellular carcinoma, and other lesions with dynamic ^68^Ga-FAPI-04 PET/CT imaging

**DOI:** 10.1186/s40658-021-00353-y

**Published:** 2021-01-22

**Authors:** Barbara Katharina Geist, Haiqun Xing, Jingnan Wang, Ximin Shi, Haitao Zhao, Marcus Hacker, Xinting Sang, Li Huo, Xiang Li

**Affiliations:** 1 Division of Nuclear Medicine, Department of Biomedical Imaging and Image-guided Therapy, Medical University of Vienna, Vienna, Austria; 2 Department of Nuclear Medicine, Chinese Academy of Medical Sciences and Peking Union Medical College Hospital, Beijing, 100730 China; 3 Beijing Key Laboratory of Molecular Targeted Diagnosis and Therapy in Nuclear Medicine, Chinese Academy of Medical Sciences and Peking Union Medical College Hospital, Beijing, 100730 China; 4 Department of Liver Surgery, Chinese Academy of Medical Sciences and Peking Union Medical College Hospital, Beijing, 100730 China

**Keywords:** Fibroblast activation protein (FAPI), Positron emission tomography, Hepatocellular carcinoma, Dual input function, Kinetic model

## Abstract

**Background:**

The study aimed to establish a ^68^Ga-FAPI-04 kinetic model in hepatic lesions, to determine the potential role of kinetic parameters in the differentiation of hepatocellular carcinoma (HCC) from non-HCC lesions.

**Material and methods:**

Time activity curves (TACs) were extracted from seven HCC lesions and five non-HCC lesions obtained from ^68^Ga-FAPI-04 dynamic positron emission tomography (PET) scans of eight patients. Three kinetic models were applied to the TACs, using image-derived hepatic artery and/or portal vein as input functions. The maximum standardized uptake value (SUV_max_) was taken for the lesions, the hepatic artery, and for the portal veins—the mean SUV for all healthy regions. The optimum model was chosen after applying the Schwartz information criteria to the TACs, differences in model parameters between HCC, non-HCC lesions, and healthy tissue were evaluated with the ANOVA test.

**Results:**

A reversible two-tissue compartment model using both the arterial as well as venous input function was most preferred and showed significant differences in the kinetic parameters *V*_ND_, *V*_T_, and BP_ND_ between HCC, non-HCC lesions, and healthy regions (*p* < 0.01).

**Conclusion:**

Several model parameters derived from a two-tissue compartment kinetic model with two image-derived input function from vein and aorta and using SUV_max_ allow a differentiation between HCC and non-HCC lesions, obtained from dynamically performed PET scans using FAPI.

## Background

Hepatocellular carcinoma (HCC), the most common primary liver cancer. HCC remains an important global clinical challenge due to its hidden onset. It is a highly heterogeneous cancer [1]. The forms of heterogeneity are seen when comparing tumors between patients (interpatient heterogeneity), between different tumor nodules within the same patient (intertumoral heterogeneity), and between different regions of the same nodule (intratumoral heterogeneity) [2]. Although imaging modalities, including ultrasonography, CT, and MRI are valuable in hepatic lesion characterization, they still have limitations in distinguishing the functional variables or the differentiation of malignant lesions [1]. There were shreds of evidence that limited sensitivity using 2-deoxy-2-[18F]fluoro-D-glucose (FDG) PET in detecting hepatocellular carcinoma with the false-negative rate approaches 40-50% [3]. Thus, the limited diagnostic efficacy of current imaging strategies remains a major challenge in the accurate evaluation of hepatic lesions.

Fibroblast activation protein (FAP) is overexpressed in cancer-associated fibroblasts (CAFs) in several tumor entities, especially in breast, colon, and pancreatic carcinomas [4]. It plays a variety of cancer-promoting roles and may predict a poor prognosis [5]. FAP-specific inhibitors (FAPIs) have already been developed as anti-cancer drugs. Recently, its quinoline-based derivatives have been designed into radiopharmaceutical agents [6, 7]. FAPI PET demonstrated promising preclinical and clinical results. A few first-in-human studies have found superior tumor-imaging potential of FAPI over FDG PET in different tumor entities [5, 6, 8, 9]. Due to low background in muscle, blood-pool and liver, the tumor-to-background contrast ratios are significantly higher. This may indicate that FAPI PET is more likely to detect tumors in the liver when FDG PET faces limitations [10].

Several investigators suggested dynamic PET with kinetic modeling might have potential value in the differential diagnosis other than static quantitative parameters like SUV [11–13]. Since the liver has blood supply from both the hepatic artery and portal vein, which should be both considered as the input function, so an accurate model is crucial for the following analysis. Few different kinetic models of FDG and ^11^C-acetate in the liver have been established. In our previous study, we compared several FDG dynamic models and introduced a simple two-tissue model using the portal vein solely to differentiate HCC from healthy liver tissues [14] accurately.

To our knowledge, the significance of FAPI PET kinetics in the liver has not been reported yet. Since the metabolic process at the cellular level is different between FAPI (binding to FAP and internalization) and FDG (entering glycolysis and accumulation), our previous liver FDG PET kinetic model [14] might not be able to fit FAPI PET kinetics. The main objective of this study, therefore, is to establish a kinetic model for ^68^Ga-FAPI-04 kinetics in distinguished hepatic lesions which were histologically determined, as well as to evaluate if the model-derived parameters allow a differentiation between HCC and non-HCC lesions non-invasively.

## Materials and methods

### Patient characteristics

The study was approved by the Ethics Committee at Peking Union Medical College Hospital. All patients signed informed consent. Eight male patients with 12 available liver lesions (age range, 47-70 years) were recruited in this study. The pathology of 7 lesions was confirmed by surgical resection and the pathology of 5 lesions was by needle biopsy. Among these patients, four patients had been histologically confirmed as hepatocellular carcinoma (HCC), two patients had intrahepatic cholangiocarcinoma, one patient had liver metastasis of gastric cardia adenocarcinoma and one had inflammatory granulomatous.

### PET/CT scan

PET/CT scans were conducted on a PoleStar m660 PET/CT scanner (SinoUnion Healthcare, Beijing, China) at Peking Union Medical College Hospital (PUMCH) [15] for all patients. CT transmission scans (120 kV, 260 effective mA) were performed first for attenuation correction and image fusion. Then, 174-259 MBq ^68^Ga-FAPI-04 was administered intravenously and the dynamic PET was performed over the liver region simultaneously. Each PET scan lasted 60 min. Dynamic PET images were reconstructed using a manufacturer (SinoUnion Healthcare, Beijing, China) who provided a stand-alone advanced research workstation with standard ordered subset expectation maximization (OSEM) algorithm with 2 iterations and 10 subsets. The 120-frame reconstruction protocol consisted of 60 frames of 5 s, 10 frames of 30 s, and 50 frames of 60 s.

### Image analysis

**Fig. 1:**
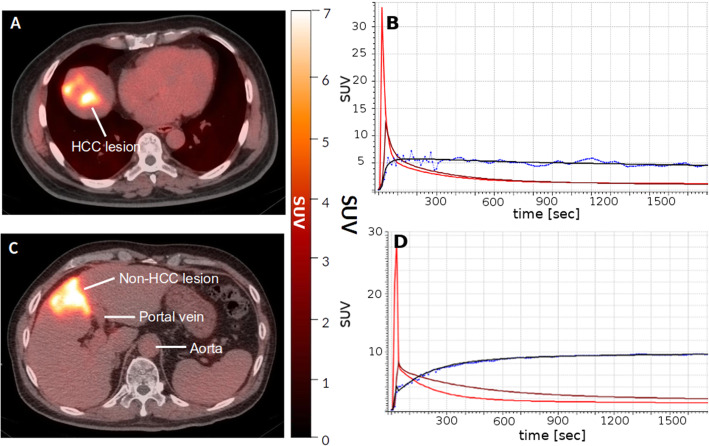
Representative image (**a**) shows HCC lesion of a 63-year-old male patient, as well as the corresponding time-activity curves (TACs) (**b**). Representative image (**c**) shows non-HCC lesion, artery, and portal vein of a 64-year-old male patient, as well as the corresponding TACs from delineated regions (**d**). For the sake of visibility, the mean SUV values in case of artery and vein are displayed

Delineation of volumes of interest was done on the Hermes Hybrid Viewer tool (Hermes Medical Solutions AB, Stockholm, Sweden). The volumes of interest (VOIs) were drawn manually over all visible lesions and healthy regions within an area far away from any lesion in the liver on the CT image of each patient. For the image-derived input functions (IDIF), a VOI was drawn within the hepatic artery (here denoted as A) and one in the portal vein (V). Then VOIs were copied to the dynamic PET images. The corresponding concentration time-activity curves (TACs) were extracted. In case of healthy regions, the mean standardized uptake value (SUV) was taken from each region. In case of the lesions and the IDIFs, the voxel with the maximum SUV (SUV_max_) was taken, since these values are least affected by partial volume and motion effects [16]. Furthermore, the according volume sizes and the uptake of the last 5 min were exported. A representative PET/CT scan is shown in Fig. 1.

### Kinetic models

**Fig. 2:**
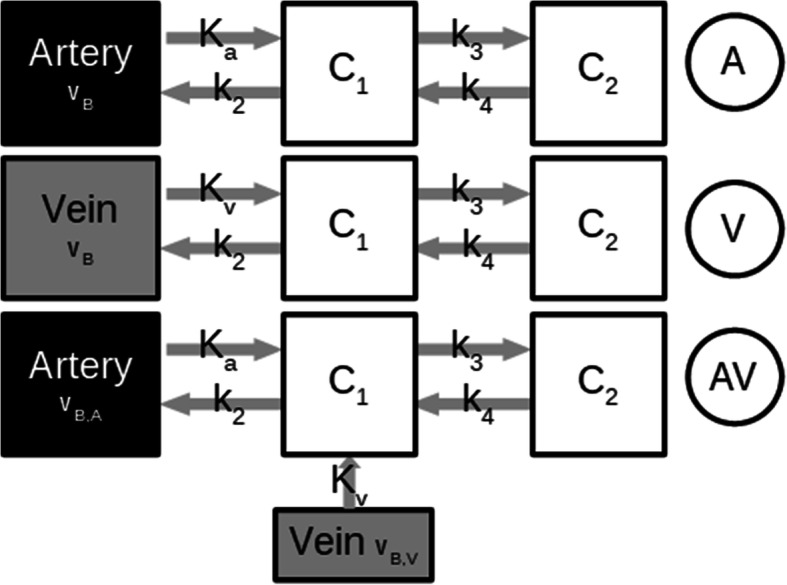
Schematic presentation of all applied models with image-derived input functions (IDIF). Model A has an IDIF from the hepatic artery, model V from the portal vein, and model AV uses both IDIFs. *v*_B_, indicates blood fraction

To describe tracer kinetic processes in a certain organ, a kinetic model is applied according to TAC. This usually is a set of differential equations that describe all underlying pharmacokinetic processes using so-called compartments, which are solved based on an input function and fitted to the TACs. As a result, rate constants (denoted with *k*) are obtained reflecting the biochemical exchange processes of the tracer. The kinetic behavior of tracers in the liver is usually described with a two-tissue compartment model using two input functions [17], since the liver tissue is supplied by both, an arterial and a venous blood input. As described above, IDIFs were used from the hepatic artery and the portal vein, both fitted with a tri-exponential function starting from the peak maximum and with a linear increase before the maximum. To find the optimum model, three different kinetic models were applied to all TACs (Fig. 2). Two reversible two-tissue compartment models were applied, as usually applied on TAC obtained from FDG PET scans: one using a single input function from the hepatic artery, denoted as “model A,” and a second one also with a single input function from the portal vein, denoted as “model V.” Additionally, a third, also with two-tissue compartmental “model AV” using both input functions from artery and vein was applied, according to the formulas:

1 $$ \frac{dC_1}{dt}={K}_aA(t)+{K}_vV(t)-\left({k}_2+{k}_3\right){C}_1(t)+{k}_4{C}_2(t) $$

2 $$ \frac{dC_2}{dt}={k}_3{C}_1(t)-{k}_4{C}_2(t) $$

with *C*_1_(*t*) and *C*_2_(*t*) as the hepatic compartment, *A*(*t*) as the artery IDIF, and *V*(*t*) as the venous IDIF. Since *A*(*t*) and *V*(*t*) are both contributing to the fraction of blood volume by having a proportion of *K*_a_/(*K*_a_ + *K*_v_) and *K*_v_/(*K*_a_ + *K*_v_) to *v*_B_ (see Eq. 3 below), each amount was assessed from their inflow rate constants for the measured compartment *C*_measured_(*t*):

3 $$ {C}_{measured}=\left(1-{V}_B\right)\left(C1(t)+{C}_2(t)\right)+{V}_B\left(A(t)\frac{K_a}{K_a+{K}_V}+V(t)\frac{K_V}{K_a+{K}_V}\right) $$

with *v*_B_ as the fraction of the measured volume occupied by blood and *K*_a_ and *K*_v_ as the influx rate constant of the aorta and the vein, respectively.

With the rate constants as fit parameters, all model fits were performed according to the least-squares method and optimized with Levenberg-Marquardt algorithm, implemented to a Java program. Errors of the fit parameters were estimated by calculating the covariance matrix. The residual sum of squares was calculated for each TAC, as well as the average and standard deviation of all rate constants; furthermore, the parameters *V*_T_ = *K*_1_/*k*_2_ (1 + *k*_3_/*k*_4_), BP_ND_ = *k*_3_/*k*_4_, and *V*_ND_ = *K*_1_/*k*_2_ were calculated, with *K*_1_ as either *K*_a_, *K*_v_ or (*K*_a_ + *K*_v_) depending on the model. Since to our knowledge no initial parameters are available for FAPI model parameters, every TAC was first fitted with a one-tissue model to obtain values for *K*_1_, *k*_2_, and *v*_B_. For the two-tissue models, these parameters were taken as initial values and a second fit was performed to obtain *k*_3_ and *k*_4_; note that the latter could become zero thus resulting in a one-tissue model for the corresponding TAC.

### Statistical analysis

In order to find the optimum model, the Schwartz criterion (SC) [18] was applied to each TAC. In detail, for each model, the number of those TACs was counted, which were preferred according to the SC and subsequently expressed as a percentage of all TACs. A Kolmogorov-Smirnov test was performed to check for normal distribution. The ANOVA test was used to find differences between HCC, non-HCC and healthy regions, significant differences between two groups were estimated with the unpaired Student’s *t* test, or the Wilcoxon-Mann-Whitney test if not normally distributed. Due to the small sample size, a *p* value of less than 0.01 was classified as significant.

## Results

### Lesion characteristics

Eight patients with 12 available liver lesions were recruited in this study. The 12 available lesions were separated according to histological results into a group of 7 HCC lesions (group HCC) and a group of 4 other lesions (2 ICC and 2 gastric metastases lesions). One lesion was an inflammation, having a TAC and uptake very similar to healthy tissue, it therefore was treated separately. Lesion characteristics are summarized in Table 1. All values showed a possible normal distribution, except for the parameters SUV, *V*_T_, and *V*_ND_ from the healthy regions.

**Table 1:** Standardized uptake value (SUV) and volume size of all investigated lesions, including the preferred model for each lesion. For the healthy region, the average overall regions is presented for SUV_mean_, size, and preferred model

Group	Lesion	SUV_max_	Size [cm^3^]	Preferred model (Schwartz criterion)
HCC	HCC-1	2.8	6.4	Model AV
	HCC-2	4.9	1.7	Model AV
	HCC-3	6.6	2.2	Model AV
	HCC-4	14.8	20.3	Model AV
	HCC-5	6.8	0.9	Model AV
	HCC-6	8.1	1.7	Model V
	HCC-7	12.9	7.0	Model A
Non-HCC	ICC-1	17.3	29.3	Model V
	ICC-2	14.8	60.7	Model A
	Metas-1	10.8	16.9	Model AV
	Metas-2	9.6	4.4	Model V
	Inflammation	1.3	4.8	Model AV
	Healthy	**SUV**_**mean**_, 0.8	40.7	Model AV

### Model selection

**Fig. 3:**
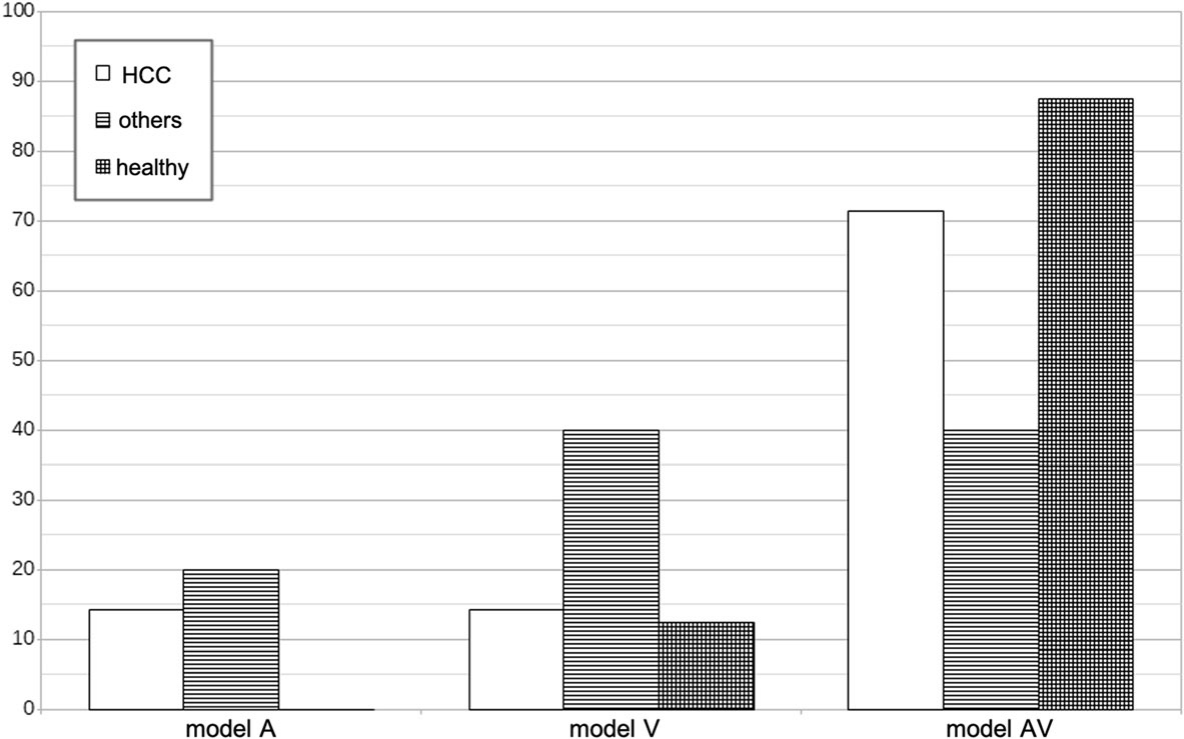
Percentage of the TACs showing a preference according to the Schwartz criterion (SC), separated for HCC lesions only, other lesions including inflammation and healthy region

For all TACs, model AV was preferred in 70% of all cases, in detail, in 58% of all lesions, in 71% of only HCC lesions, and in 88% of all healthy regions, see Fig. 3. This distribution did not change after excluding the inflammation lesion. All relevant mean parameters derived from model AV are listed in Table 2 for all groups separately. With only one exception, *k*_3_ and *k*_4_ were zero in the case of all healthy regions.

**Table 2:** Results for the relevant obtained model parameters of model AV for all lesions and healthy regions. The *p* value for the ANOVA test for all four groups is also given, results < 0.01 are underlined. Values are presented as mean value plus-minus one standard deviation. Note BP_NP_ is *n* presented for healthy regions since *k*_3_ and *k*_4_ were zero or almost zero

	HCC lesions	Non-HCC lesions	Inflammation	Healthy	ANOVA test
*K*_a_ [min^−1^]	0.5 ± 0.3	0.3 ± 0.3	0.0	0.5 ± 0.3	0.02
*K*_v_ [min^−1^]	0.8 ± 0.7	0.4 ± 0.5	1.6	0.6 ± 0.7	0.23
*k*_2_ [min^−1^]	1.6 ± 0.7	0.6 ± 0.2	3.6	1.5 ± 0.6	0.01
*k*_3_ [min^−1^]	0.11 ± 0.03	0.21 ± 0.09	0.01	0.01 ± 0.01	0.12
*k*_4_ [min^−1^]	0.05 ± 0.04	0.04 ± 0.02	0.03	0.01 ± 0.01	0.33
*K*_i_	0.10 ± 0.02	0.19 ± 0.09	0.08	0.01 ± 0.03	0.0002
*V*_T_	3.7 ± 1.4	7.6 ± 1.5	4.7	0.06 ± 0.06	< 0.0001
*V*_ND_	0.9 ± 0.4	1.2 ± 0.3	1.0	0.8 ± 0.2	< 0.0001
BP_ND_	3.2 ± 1.3	5.6 ± 0.6	0.44		

### Comparison between HCC and non-HCC

**Fig. 4:**
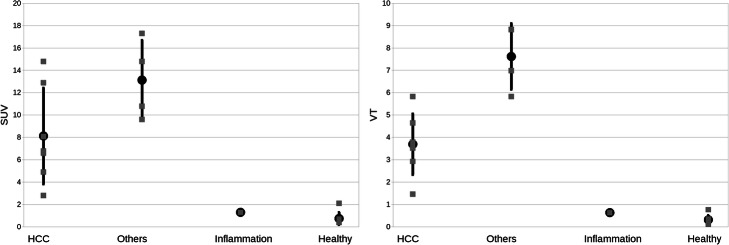
For HCC lesions, non-HCC lesions, one inflammation, and healthy regions, the SUV uptake for the last 5 min (left) as well as the parameter *V*_T_ derived from model AV (right) are displayed. Differences between HCC and non-HCC lesions were not significant in case of the SUV uptake (averaged 61 % difference, *p* = 0.08), and significant in case of *V*_T_ (averaged 106% difference, *p* = 0.002)

No significant differences were found in the SUV uptake according to the ANOVA test between all different groups HCC, non-HCC, inflammation and healthy (*p* = 0.06), nor between only HCC and non-HCC group (8.1 versus 13.1, *p* = 0.08), see Fig. 4. However, comparing only the SUV uptake from all lesions together (HCC and non-HCC) versus the healthy regions, a significant difference was found (9.9 versus 0.8, *p* = 0.0002 according to both, Student’s *t* test and Wilcoxon-Mann-Whitney test). Including the inflammation lesion to the lesion group, the difference was slightly less significant with *p* = 0.0001 (9.9 versus 0.8). Regarding volume sizes, no significant differences were found between HCC and non-HCC groups without the inflammation (6 cm^3^ versus 23 cm^3^, *p* = 0.09).

Note that for all applied models, the differences in *k*_3_ and *k*_4_ were always significant between HCC and non-HCC lesions compared to healthy regions, since *k*_3_ and *k*_4_ were equal or close to zero for all models and all healthy regions. For models A and V, there were no significant differences found between HCC and non-HCC lesions in case of all rate constants and the macro-parameters *V*_T_ (*p* = 0.08 and *p* = 0.10, for model A and V, respectively) and *V*_ND_ (*p* = 0.73 and *p* = 0.57). In case of BP_ND_, a significant difference was found only for model A: 2.9 for HCC and 6.4 for non-HCC, *p* = 0.0006.

With model AV, significant differences according to the ANOVA test were found between all groups in case of the parameters *V*_T_ (*p* < 0.0001), *V*_ND_ (*p* < 0.0001), and BP_ND_ (*p*< 0.0001). Including the inflammation, lesion did not change this finding (all according *p* < 0.001). Comparing all groups among each other without healthy regions (which had *k*_3_ and *k*_4_ of almost or equal zero), a significant difference was also found in BP_ND_ (*p* = 0.003). A direct comparison of HCC and non-HCC lesions (without inflammation) showed significant differences in *V*_T_ (3.7 versus 7.6, *p* = 0.002), *V*_ND_ (0.9 versus 1.2, *p* = 0.29), and BP_ND_ (3.2 versus 5.6, *p* = 0.006).

## Discussion

Noninvasive assessment of liver mass is crucial in differentiating hepatic malignancy. Many cases are already advanced at the time of diagnosis since the occult onset and lack of effective biomarkers for hepatic malignancy [19, 20]. Our findings showed preliminary results that using dynamic FAPI PET with kinetic modeling could identify HCC from a variety of hepatic tumors. It suggested the dynamic FAPI PET imaging might have the potential to allow a precise determination of hepatic malignancy noninvasively.

However, there were no reliable corrections for partial volume or motion effects available; therefore, the values in particular for *K*_a_, *K*_v_, and *k*_2_ may not be taken as real values with biological meaning. Although these effects are minor in the case of the aorta [21, 22], this is certainly not the case for smaller blood vessels like the portal vein. However, to reduce these effects, SUV_max_ was taken for all IDIFs [16], and also for all lesions due to their partially very small sizes.

Although only minor knowledge is known of FAPI kinetics, it seemed obvious to choose two-tissue compartmental models, since FAPI apparently was trapped in the organ and the lesions. We assume that in case of all lesions, a one-tissue compartmental model (i.e., *k*_3.4_ = 0) was not sufficient, because the fits resulted in values of *k*_3,4_ > 0 for all lesions are values of *k*_3,4_ ≈ 0 for all healthy regions.

Not surprisingly, the preferred model was a model taking venous and arterial input into account, since liver tissue in general is supplied by both [17]. Using this model, significant differences could be found between the four investigated groups, HCC, non-HCC, and healthy regions in case of all rate constants, the binding potential BP_ND_, the total distribution volume *V*_T_, and distribution volume of non-displaceable uptake *V*_ND_. Note that the results were not changed significantly after including the inflammation lesion into this analysis. These results therefore suggest that there are kinetic differences in FAPI kinetic between HCC and non-HCC lesions. Also not surprising, *k*_3_ and *k*_4_ were zero for almost all healthy regions, meaning that a reversible one-tissue model described healthy tissue best.

The inflammation lesion was treated separately since its SUV uptake as well as its curve shape was very similar to healthy tissue having also similar model parameters. A wrong delineation aside from the real lesion due to motion effects can be excluded: a shifting of PET data due to motion effects would most probably affect the whole organ; thus, similar curve shapes due to wrong delineations would have been observed also in other, even smaller lesions, which was not the case. Thus, if the delineation was correct, we conclude that FAPI tracer kinetics might be similar to healthy regions in case of inflammation.

A major limitation of this study certainly is the small cohort size. However, this was a first proof-of-concept study showing that IDIFs (with a simple compensation method) are sufficient to derive relevant model parameters from describing FAPI kinetics in liver lesions. Also, the lesions are very heterogeneous, in particular, the one inflammatory lesion was difficult to handle. Therefore, it was treated separately and the analysis was done twice, with and without including the inflammation. Moreover, due to a missing reliable motion and partial volume corrections, values like *K*_1_ and *k*_2_ cannot be taken as biologically relevant; however, the macro parameters V_ND_, BP_ND_, and mainly V_T_ show significant differences between all groups. Further studies, with a reliable integrated correction method and including a comparison to pathological results, will be conducted.

## Conclusion

In the present study, we investigated kinetic models for FAPI PET in liver lesions, showing that the consideration of the maximum SUV values for artery and venous image-derived input function is suitable to at least distinguish between healthy regions, HCC lesions, and non-HCC lesions in model-derived macro parameters.

## Data Availability

Please contact the corresponding author for data requests.

## References

[1] Craig AJ, von Felden J, Garcia-Lezana T, Sarcognato S, Villanueva A (2020). Tumour evolution in hepatocellular carcinoma. Nat Rev Gastroenterol Hepatol..

[2] Nault JC, Villanueva A (2015). Intratumor molecular and phenotypic diversity in hepatocellular carcinoma. Clin Cancer Res..

[3] Ho CL, Yu SC, Yeung DW (2003). 11C-acetate PET imaging in hepatocellular carcinoma and other liver masses. J Nucl Med..

[4] Siveke JT (2018). Fibroblast-activating protein: targeting the roots of the tumor microenvironment. J Nucl Med..

[5] Giesel FL, Kratochwil C, Lindner T (2019). (68)Ga-FAPI PET/CT: biodistribution and preliminary dosimetry estimate of 2 DOTA-containing FAP-targeting agents in patients with various cancers. J Nucl Med..

[6] Loktev A, Lindner T, Mier W (2018). A tumor-imaging method targeting cancer-associated fibroblasts. J Nucl Med..

[7] Lindner T, Loktev A, Altmann A (2018). Development of quinoline-based theranostic ligands for the targeting of fibroblast activation protein. J Nucl Med..

[8] Giesel FL, Heussel CP, Lindner T (2019). FAPI-PET/CT improves staging in a lung cancer patient with cerebral metastasis. Eur J Nucl Med Mol Imaging..

[9] Kratochwil C, Flechsig P, Lindner T (2019). (68)Ga-FAPI PET/CT: tracer uptake in 28 different kinds of cancer. J Nucl Med..

[10] Shi X, Xing H, Yang X, Li F, Yao S, Zhang H, Zhao H, Hacker M, Huo L, Li X. Fibroblast imaging of hepatic carcinoma with 68Ga-FAPI-04 PET/CT: a pilot study in patients with suspected hepatic nodules. Eur J Nucl Med Mol Imaging. 2020. 10.1007/s00259-020-04882-z 32468254

[11] Park JW, Kim JH, Kim SK (2008). A prospective evaluation of 18F-FDG and 11C-acetate PET/CT for detection of primary and metastatic hepatocellular carcinoma. J Nucl Med..

[12] Huo L, Guo J, Dang Y (2015). Kinetic analysis of dynamic (11)C-acetate PET/CT imaging as a potential method for differentiation of hepatocellular carcinoma and benign liver lesions. Theranostics..

[13] Okazumi S, Isono K, Enomoto K (1992). Evaluation of liver tumors using fluorine-18-fluorodeoxyglucose PET: characterization of tumor and assessment of effect of treatment. J Nucl Med..

[14] Geist BK, Wang J, Wang X, Lin J, Yang X, Zhang H, Li F, Zhao H, Hacker M, Huo L, Li X. Comparison of different kinetic models for dynamic 18F-FDG PET/CT imaging of hepatocellular carcinoma with various, also dual-blood input function. Phys Med Biol. 2020;65:045001. 10.1088/1361-6560/ab66e3 31896098

[15] Huo L, Li N, Wu H (2018). Performance evaluation of a new high-sensitivity time-of-flight clinical PET/CT system. EJNMMI Phys..

[16] Soret M, Bacharach SL, Bivat I (2007). Partial-volume effect in PET tumor imaging. JNM..

[17] Munk OL, Bass L, Roelsgaard K, Bender D, Hansen SB, Keiding S (2001). Liver kinetics of glucose analogs measured in pigs by PET: importance of dual-input blood sampling. J Nucl Med..

[18] Golla SSV, Adriaanse SM, Yaqub M (2017). Model selection criteria for dynamic brain PET studies. EJNMMI Phys..

[19] Omata M, Cheng AL, Kokudo N (2017). Asia–Pacific clinical practice guidelines on the management of hepatocellular carcinoma: a 2017 update. Hepatol Int.

[20] Margini C, Dufour JF (2016). The story of HCC in NAFLD: from epidemiology, across pathogenesis, to prevention and treatment. Liver Int..

[21] Germano G, Chen BC, Huang SC, Gambhir SS, Hoffman EJ, Phelps ME (1992). Use of the abdominal aorta for arterial input function determination in hepatic and renal PET studies. J Nucl Med..

[22] Geist BK, Baltzer P, Fueger B (2018). Assessing the kidney function parameters glomerular filtration rate and effective renal plasma flow with dynamic FDG-PET/MRI in healthy subjects. EJNMMI Res..

